# 

**Published:** 1861-04

**Authors:** 


### CONTENTS.

ORIGINAL ESSAYS AND COMMUNICATIONS.

Human Knowledge,—Natural and Supernatural. By B. P. Aydelott, D. D. .......... 198
Plates over Fangs. By W. H. Atkinson, .......... 213
Who are Dentists? By W. A. Pease, .......... 216
Alveolar Abscess,—Treatment and Cure. By W. H. Shadoan, .......... 221
A New Napkin Holder. By Wm. A. Pease, .......... 225
Formation of Local Societies, .......... 226

PROCEEDINGS OF SOCIETIES.

Seventeenth Annual Meeting of the Mississippi Valley Association, .......... 227

SELECTIONS.

Block Teeth, .......... 239
The Extraction of Teeth, .......... 243

CORRESPONDENCE.

A Letter from Philadelphia .......... 252
Mad River Valley Society, .......... 254
Editorial .......... 255

